# Destruction of Fly Larvae in Horse Manure

**Published:** 1915-07

**Authors:** 


**Destruction of Fly Larvae in Horse Manure.** F. C. Cook, Bull. No. 118 U. S. Dept. of Agriculture, states that the following have been found inefficient or only partially satisfactory: kerosene emulsion, kainit, Isthmian Canal Commission larvicide, ferrous sulphate, potassium cyanid, formaldehyde, Calcium cyanamid, pyroligneous acid, sodium chlorid, copper sulphate, lime-sulphur mixture, Paris green, sodium fluorid, ammoniacal gas liquor, and several proprietary mixtures. Borax and calcined colemanite proved satisfactory, the former being preferred on account of general convenience. 0.62 pound borax or 0.75 colemanite is used to every 10 cubic feet or 8 bushels of manure, applied with a sieve and especially thick to the outer edges of the pile. 2-3 gallons of water are then sprinkled over the pile. Such treatment should be applied promptly to prevent hatching of eggs. Borax may also be sprinkled about stables. The cost is estimated at a cent a day per horse. (Note: Measures of this kind are infinitely more efficient and safe than swatting. The supplanting of horses with automobiles in large areas of the residence districts of cities has had a favorable influence in a negative way and because the odor of gasoline repels flies and other insects).

